# Supplementary material for: GJB2 Is a Major Cause of Non-Syndromic Hearing Impairment in Senegal
Source: Biology (Basel). 2022 May 23;11(5):795. doi: 10.3390/biology11050795 (PMC9138795; doi:10.3390/biology11050795)
Supplement: Supplementary file 1 [file biology-11-00795-s001.zip › biology-1722585-supplementary.pdf]

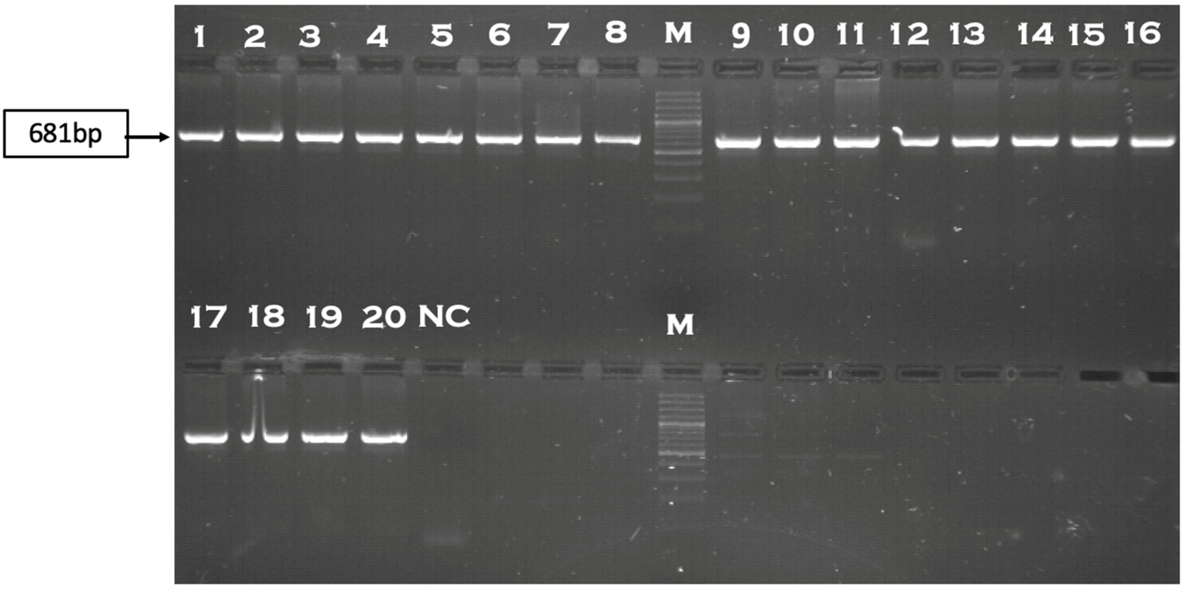

Figure S1. PCR-Multiplex products visualized on 2% agarose gel. We obtained a PCR product of 681 bp corresponding to the wild type (GJB6-1R→GJB6-2R).
M and NC indicate the Molecular weight and Negative Control respectively.

Table S1. Variant's interpretation according to the ACMG guidelines

ACMG criteria		
Variants		Clinical significance
c.94C>T	PP3_Supporting, PM2_Strong, PM1_Strong, and PP5_Strong	Pathogenic
c.427C>T	PP5_Very strong, PM5_Moderate, PM2_Supporting, PP2_Supporting and PP3_supporting	Pathogenic
132G>A	PVS1_Very strong, PM2_Strong, PP5_Strong and PP3_Supporting)	Pathogenic
